# Bereavement and support in the conduct of everyday life: Insights from dual experiences of loss and drug use

**DOI:** 10.1177/14550725251329826

**Published:** 2025-04-10

**Authors:** Lillian Bruland Selseng, Sari Kaarina Lindeman, Monika Alvestad Reime

**Affiliations:** 1657 Western Norway University of Applied Sciences, Sogndal, Norway; 1657 Western Norway University of Applied Sciences, Bergen, Norway; 1657 Western Norway University of Applied Sciences, Sogndal, Norway

**Keywords:** Bereavement support, companionship, complicated grief, drug use, conduct of everyday life, qualitative interviews, sociality, three-tiered support model, trust

## Abstract

**Aims:** Individuals with a history of heavy drug use are especially vulnerable to losing loved ones to drug-related deaths, and drug use and complicated grief reactions are closely linked. However, limited knowledge exists on supporting them through bereavement. The present study, which was conducted in Norway, aims to provide a comprehensive understanding of how individuals dealing with heavy drug use and bereavement can be supported through professional support services. The bereaved person's reflections on help experiences are seen as grounded in their conduct of everyday life. **Methods:** We conducted qualitative interviews with individuals who have experienced bereavement in the context of heavy drug use. Using reflexive thematic analysis, we examined their stories and reflections on what was helpful and unhelpful, aiming to identify key themes, providing an understanding of how individuals dealing with heavy drug use and bereavement can be supported. **Results:** Five themes were identified. The first theme, ‘Support Complexity’, addresses the uncertainty about strengthening the help provided for processing losses. The other themes – “The Ripple Effect of Trust and Distrust”, “Offering Supportive Communication”, “Fostering Tailored Support” and “Promoting Support Through Companionship” – point to the key dimensions of the support that can help strengthen responses to drug-related bereavement. **Conclusions:** The study underlies how diverse expectations, norms and positions in the help provision setting influence the nature of support, as well as the access to, acceptance of and adaption to support initiatives. Furthermore, we discuss how the support context can provide multifaceted and contextualised bereavement support tailored to the various needs of the bereaved. The study also underscores the need for provider training focused on the intersections of bereavement and drug use.

## Introduction

This study explores the bereavement support needs of individuals who have experienced drug-related deaths in the context of heavy drug use. Building on the emphasis on the sociality of human subjectivity of Dreier (2009, 2011), we explore and illuminate their desires for support services within the context of their everyday practices and prevailing perceptions of helping practices. The study highlights the perspectives of individuals with heavy drug use who have lost family members, intimate partners or close friends to drug-related deaths. By emphasising the importance of social context, we explore, from a first-person perspective, the bereavement support needs of these individuals. The study aims to provide a more extensive understanding of how individuals who live with heavy drug use and bereavement can be supported through professional support services.

### Bereaved drug users: a complex life situation

Previous research on the social context of drug use has underlined that people with heavy drug use often have poor physical and mental well-being. Individuals with heavy drug use may also face additional challenges such as homelessness, poverty, unemployment (Brekke et al., 2018), and social isolation and loneliness (Ingram et al., 2020). Individuals with heavy drug use are as diverse as any other group of individuals, yet there is a significant overlap between mental health issues and drug-related challenges (Toftdahl et al., 2016). Research has shown that difficult life experiences can affect the forming and maintenance of relationships with service providers and the social network (Heleniak et al., 2016).

Heavy drug use is known to pose a high risk of injury and premature death (European Monitoring Centre for Drugs and Drug Addiction, 2024). Approximately 600,000 individuals die in drug-related deaths every year worldwide, impacting millions of bereaved people (World Health Organization, 2024). In Norway, the number of overdose deaths in 2023 was the highest in years, with a total of 388 overdoses (Norwegian Institute of Public Health, 2024). In addition to these, there are drug-related deaths resulting from accidents, suicides, homicides and somatic diseases. Generally speaking, such unnatural deaths are found to cause severe and long-lasting health-related and social consequences for those close to the deceased, with an increased risk of prolonged grief disorder (Djelantik et al., 2020; Titlestad & Dyregrov, 2024).

Being a participant in a social context where people's daily lives are marked by heavy drug use often means having relationships with others who are also engaged in heavy drug use. This places individuals in such social contexts at particularly high risk of witnessing a drug-related death and losing loved ones to such deaths (Kenny et al., 2022; Schlosser & Hoffer, 2022; Wojtkowiak et al., 2019).

The social context of heavy drug use also influences the bereavement process. Previous research has shown that the loss of close persons is often associated with an increase in drug use (Macmadu et al., 2022; Masferrer et al., 2015; Schlosser & Hoffer, 2022; Selseng et al., 2023). Drug use is perceived as a way of managing feelings of bereavement and trauma (Schlosser & Hoffer, 2022; Selseng et al., 2023). Previous studies also indicate that drug-related deaths can not only lead to an altered view of death, but also they can become a transformative life event (Selseng et al., 2023; Wojtkowiak et al., 2019). Individuals who have relied on drugs to cope with death and loss may experience heightened emotions and difficulties to cope without drugs in these types of situations (Selseng et al., 2023, 2024). A systematic review investigating the relationship between complicated grief and drug use showed that bereaved who use drugs are at higher risk than others of complicated grief reactions (Parisi et al., 2019).

As a result of heavy drug use, these individuals may withdraw from others. Likewise, those around these individuals may also withdraw due to their drug use (Caparrós & Masferrer, 2021; Selseng et al., 2024; Wojtkowiak et al., 2019). This social withdrawal may hinder access to social support, which research has shown to be crucial in preventing complicated grief (Reime, O’Connor et al. 2024b; Titlestad & Dyregrov, 2024). Others can start to focus on reducing the drug use of these individuals rather than acknowledging them as bereaved individuals (Selseng et al., 2024; Selseng et al., 2023). Previous studies indicate that bereaved individuals who use drugs often avoid social events and rituals related to death in attempts to shield themselves from multiple deaths, practical difficulties and feelings of being unwelcome (Schlosser & Hoffer, 2022; Selseng et al., 2024).

Relationships with others who also heavily use drugs may create a social context where expressing emotions or experiences related to grief is uncommon. Instead, a culture that appears to promote toughness and the suppression of feelings may be established (Macmadu et al., 2022; Schlosser & Hoffer, 2022; Selseng et al., 2024; Wojtkowiak et al., 2019). Overall, an individual who engages in heavy drug use may experience multiple losses, live in a tough environment characterised by silence and loneliness, and hold a little-recognised position as a bereaved person. These distinct contextual factors related to bereavement make it necessary to develop awareness and knowledge on how the contributions of professional support services can be optimised when working with this group of bereaved individuals.

Offering bereavement support involves understanding and navigating the social contexts in which individuals who engage in heavy drug use participate. An illustrative example of the importance of considering social context is provided by Moore (2004). Moore points out that overdose prevention strategies based on assumptions of rationality and personal autonomy often misalign with the social, cultural and economic realities of street-based individuals who inject drugs. This example underscores the need for interventions to be attuned to the social context and conduct of everyday life of those they aim to help.

In addition, the support required for individuals using drugs becomes more complex when they have multiple simultaneous needs for assistance (Savic et al., 2017). When individuals with heavy drug use experience losses, this can generate uncertainty among helpers (Løseth et al., 2022; Reime et al., 2024a; Totland et al., 2024). Previous research indicates that professionals who are familiar with heavy drug use seek more competence in bereavement support, while those experienced in bereavement support feel less certain when dealing with heavy drug use (Løseth et al., 2022; Reime et al., 2024a; Totland et al., 2024). Individuals with heavy drug use may also have difficulties accessing services (Cortis, 2012). Barriers to help provision and help seeking can also be understood in light of the theory of access (Schaffer & Wen-hsien, 1975), which highlights aspects within public bureaucracies that can lead to the unequal distribution of services. For example, the conditions for admission, ordering and encounters influence the provision and reception of support.

A systematic review of how family members experience drug-death bereavement showed that a lack of understanding and help from support systems as well as stigmatisation made life situations and processing the bereavement demanding (Titlestad et al., 2021a). The results indicate that the bereaved need tailored, integrated help and support, as well as coherent user pathways (Titlestad et al., 2021a). Another study examining drug-death bereaved persons’ advice to policymakers on how to improve bereavement services after a drug-related death highlights the importance of services that are routinised and have a broad-spectrum, ensuring that help is provided in a respectful and competent way (Fjær & Dyregrov, 2021).

Although situations involving heavy drug use and bereavement is a challenging and risk-laden life situation for the affected individual and a demanding context for helpers, there is a lack of knowledge about the best ways to provide assistance in such difficult circumstances (Caparrós & Masferrer, 2021; Masferrer et al., 2017; Selseng et al., 2024). The combination of life stressors and illicit drug use not only increases the risk of complicated grief reactions, but also poses challenges in obtaining appropriate help and support.

### Theoretical framework: conduct of everyday life

Societies are structured around and consist of various contexts in which social life progresses and is made sense of. Just as social entities and organisations such as workplaces and schools have their own social order of practices with formal and informal rules, so do the social contexts in which people use drugs. In this ontological outlook, social contexts are socio-material units through which various social practices (e.g., such as work, education, health care and drug use) are conducted. In his theorisation on social contexts, psychologist Ole Dreier has emphasised that arrangements of social contexts define who qualifies as legitimate participants, establish specific social positions and delineate particular expectations and responsibilities for the persons who participate in their social practices (Dreier, 2011).

People manage their lives through their conduct of everyday life in relation to the social arrangements they participate in. The conduct of everyday life entails individuals coordinating their diverse obligations, relationships and activities with their co-participants across various social contexts throughout the day. The order of everyday life and the specific social contexts that individuals participate in influence how they live their everyday lives, shaping their perspectives on life, on themselves and on others, influencing the functioning of the person. (Dreier, 2009, 2011). As such, persons are always situated within a social context from where their perspectives of experience and their activities reach out.

For those whose drug use is an essential part of how they conduct everyday life, various social practices related to drug use, such as legislation and dominating cultural norms, may influence their daily interactions and behaviours (Duff, 2007; Measham & Shiner, 2009; Shiner & Newburn, 1999). The social contexts they participate in situate their activities and their psychological processes This theoretical framework of the conduct of the everyday life helps us understand the complexities of bereavement support among individuals with a dual experience of loss and heavy drug use. In the present study, it is used to comment on and discuss the results, providing deeper insights into the data.

### The Norwegian context

The context of the research is Norway, characterised by developed welfare schemes, comprehensive public social policies and universal social rights (Fernández-Alonso & Jaime-Castillo, 2016; Kildal, 2013; Titlestad et al., 2021b). Services for individuals with heavy drug use over time face a complex array of challenges, as regularly documented in government evaluation reports. These reports highlight substantial variations in wait times, assessment, treatment and follow-up care (Ådnanes et al., 2021). Individuals requiring long-term assistance from various services encounter support systems within distinct social contexts. This involves services specifically related to drug use, such as treatment and follow-up care, with specialised expertise in heavy drug use. Furthermore, it includes broader health and social services available to the general population, such as those provided by the Norwegian Labour and Welfare Administration (NAV) or general practitioners without specialised competencies in drug use. As a result, the knowledge and expertise regarding heavy drug use can vary significantly among service providers. Different understandings of heavy drug use arise across sectors and disciplines, with stakeholders possessing varied experiences and perceptions of how drug use can present a problem that impacts everyday practices (Helsedirektoratet, 2024). This diversity in social contexts and professional competencies poses a significant challenge in delivering cohesive and comprehensive support to this group (Helsedirektoratet, 2024). This enduring challenge underscores the need for better coordination and integration among different service providers to ensure individuals receive the consistent and specialised care they need, although this remains a persistent challenge for the services (Ådnanes et al., 2021). In evaluations of substance use services in Norway, the importance of the relationship with service providers is evident (Fafo, 2021; Hansen et al., 2020). How individuals are met, listened to and recognised in their interactions with the service system significantly impacts their experiences and outcomes. Moreover, assistance in breaking isolation and gaining access to a satisfactory social network are highly valued by individuals receiving support. The evaluations highlight the necessity of focusing on the content of the services offered, ensuring they are tailored to meet their needs and are accessible when required (Fafo, 2021; Hansen et al., 2020).

## Methods

The present study is based on data from a larger study on bereavement after drug-related deaths in a recovery perspective in Norway (the END project). The END project addresses bereavement from the perspectives of different groups of bereaved persons (parents, siblings and close friends), as well as from the helpers’ perspective and uses a mixed method design.

The present article used semi-structured interviews with 13 individuals who had a history of heavy drug use and had lost a close friend, partner or ex-partner due to drug-related deaths. All participants had experiences of illicit drug use, and all of them had either prior experiences of or current contact with public services related to their drug use. Seven women and six men participated in the interviews. Four of the women lost an intimate partner, of whom one had lost the father to her child. Three of the female participants had lost a friend, of whom one was also the father to her child. Five of the men had lost a friend and one man had lost his ex-girlfriend/friend who was also the mother to his child. The age of the research participants varied from 21 to 54 years, and the time since the death varied from some months to 30 years. Moreover, several of the participants had experienced multiple losses. The context of the death and the participants’ situation at the time of the death varied. Some of the participants (*n* = 2) were present at the time of the death (overdose), others were in treatment, some were not using drugs at the time of the death and others were actively using drugs. At the time of the interviews, two participants were receiving drug use treatment, two were actively using drugs, eight reported having recovered from drug use and one used drugs occasionally.

Six of the bereaved individuals were recruited from a survey sample (*n* = 255), in which they reported that they were willing to be contacted for individual interviews. The survey was administered in 2018 and included varied validated instruments to measure bereavement experiences and the health and social consequences, in addition to descriptive variables (i.e., time since loss, relation to the diseased, age of diseased, own drug use problems). To increase the sample size, an additional seven bereaved individuals with a history of heavy drug use and bereavement were recruited by various methods, including the snowballing method, through the researchers’ professional networks and by information to municipalities, non-governmental organisations and treatment centres. Individuals undergoing treatment or actively using drugs may find it challenging to participate in a research study due to their complex life situations. Through snowball sampling, participants received information from people within their professional or personal networks who encouraged and supported them. This support appeared to be crucial for their participation. However, it also implied that individuals without similar support networks might have been unable to engage in the research.

The interview participants were informed about the aims and methods of the study and their rights as research participants, including their rights to withdraw from the study at any time. An informed consent was filled in.

The 13 semi-structured interviews were conducted between July 2019 and October 2020 in places such as peer organisations, hotel lobbies, public offices and treatment centres. The interviews followed a thematic interview guide with themes exploring the time before death, experiences of loss, the time after death, experiences with stigma and experiences with help and support. The first and second researchers conducted all interviews except for one, which was conducted by another researcher working on the project. The interviews were designed to be dynamic and respondent focused. The interviews were based on a common thematic guide, but what was elaborated upon and focused on could vary depending on what the participants shared. For example, some of the research participants were asked directly if they had some advice on how to improve support in these situations, while others were not asked directly about advice but made the theme relevant when they talked about their experiences with help and support. The interviews lasted 1–2 h and were audio recorded. To talk about the loved one and the loss can be hard as well as provoke memories and thoughts that have been suppressed for years. The researchers were conscious of any reactions from the research participants that would need further follow-up in the aftermath of the interview. After the interview was finished, the researchers asked for participants’ experiences of the interview setting and, eventually, the need for a follow-up. In situations where the researchers sensed that the situation could be challenging, the researcher followed up with a telephone call to the research participant and/or provided information about relevant bereavement support services. The study followed ethical guidelines and was approved in February 2018 by the Norwegian Regional Ethical Committees for Medical and Health Research Ethics (2017/2486/REK vest).

The data was analysed using reflexive thematic analysis, a family of methods capturing the core meaning of what is shared, emphasising researcher subjectivity and interpretative practice (Braun & Clarke , 2021, 2023). In this, a theme represents the core ideas and arguments under a particular concept. Themes respond to the purpose and research query. The first author conducted the analysis, while the other researchers, who had also read the interviews, contributed to ongoing discussions and reflections. The analysis was guided by the presumption that the participants had valuable experiences and insights regarding what support had been helpful for them, or could have been helpful, in the context of their substance use and the loss of a loved one due to drug-related deaths. We specifically looked for statements that provided such insights during the coding process. The theoretical framework was introduced after the analysis was completed.

In the first phase, all the interviews were read, and the relevant reflections and questions were noted. In the second reading, the statements which were related to support for drug-death bereavement, both explicit and implicit through stories from experiences with help, were identified. Then, they were coded through a thorough close reading of the marked texts. The codes aimed to capture the core meaning of what was shared. These codes were then grouped into themes based on the united meaning of the codes. The process of coding and developing themes involved going back and forth between the themes and the interview data. This work was presented, and alternative interpretations were discussed with the other researchers. Interim themes were also discussed with experts-by-experiences who participated in the research project.

Finally, based on consensus in the research team, five themes were developed to describe the overall meaning-based interpretive stories of the data, even though the themes partially overlap: (a) Support Complexity, (b) The Ripple Effect of Trust and Distrust, (c) Offering Supportive Communication, (d) Fostering Tailored Support and (e) Promoting Support Through Companionship. After the themes were written up, we discussed which theoretical lens could illuminate the findings in a relevant and knowledge-enhancing way. For reasons accounted for earlier in this article, we chose the description of the conduct of everyday life by Dreier (2009, 2011) and reflected the results in light of this perspective.

### Findings

In this section, we present the five themes related to understanding how individuals dealing with heavy drug use and bereavement can be supported. The themes are each presented with illustrative quotes which have been anonymised and assigned fictitious names.

#### Support complexity

Based on the interviews it stood clear that only a few participants had experienced professional help that was beneficial for their bereavement process. When we invited reflections on whether anyone or anything could have helped them in their situation after the death of their close one, many expressed uncertainties. We often received responses such as “I don't know” and “I’m not sure”. Several of the participants described themselves as being difficult to help in this situation. They pointed to their use of drugs as a means to try to cope with or reduce the pain of their loss. Consequently, they perceived themselves as less accessible and less motivated to seek out and embrace professional help. One of those who expressed this was Anders. He put it this way: “I wasn't willing to accept help in my grief. I wasn't willing to listen to anyone”.

The way in which Anders framed this utterance indicated that accepting professional help would have required him to make significant changes to how he conducted his everyday life, which he was not willing to do. The uncertainties expressed by several individuals about whether and how professional help could have been beneficial imply that they perceived professional help as quite different from the social contexts of their everyday lives. By positioning themselves as “difficult to help”, they express a self-understanding in which they do not see themselves as possessing the skills or characteristics necessary to be helped or being receptive to help. Their drug use is portrayed as a central activity, creating a gap between their own life-world context and the social context of professional help.

Several individuals also indicated their uncertainty about the potential benefits of help, citing a lack of past experiences to draw upon. They shared that, since no one had ever attempted to provide support, they couldn't assess whether it could be helpful. Bjørn expresses it like this:

It's primarily drugs [that we turn to] because who are we supposed to talk with then? Where are we supposed to go? There isn't anything, and there isn't much in the way of support from the welfare system or support from anyone to go and talk with a psychologist about it. There isn't a system for that. So, then you end up dealing with it through drugs very often. (Bjørn)

When Christine reflects on the lack of support after the death of her loved one, she also finds it difficult to know what might have helped since no one had tried. At the same time, she describes an irritation over the fact that no one had tried:

Maybe I would have told them to get lost, but perhaps I wouldn't have been so irritated afterwards when I think about these things because I notice that I get engaged in it. So, then I wonder, why the hell didn't I get any offer about anything at all? There was nothing. (Christine)

The questions that Bjørn asked about, regarding where to go and whom to talk to, indicate that he perceives professional bereavement support as foreign to and inaccessible in his conduct of everyday life. There is a perceived lack of support services that work alongside and interact with the social contexts of their everyday lives.

Christine's irritation suggests an expectation that support service should be available to them. Her frustration of not being offered anything demonstrates the significance of being offered help, even if it goes unused. Receiving an offer, even if it is not accepted, may give the bereaved a feeling of being seen and acknowledged, which can positively impact the subsequent bereavement process.

#### The ripple effect of trust and distrust

The next theme addresses the critical role that previous interactions with health and welfare services play in shaping the bereaved individual's openness to bereavement support. Many participants reflected on how their experiences with these institutions prior to the loss had significantly influenced their willingness and capacity to engage with help and support related to their bereavement.

For example, Dina had established a trusting relationship with some staff members at the residential treatment facility that she attended for a lengthier period. When she lost her intimate partner to an overdose, she left the treatment facility and entered a phase of intensive drug use, but the relationship she had with the staff continued. She trusted them and felt that they recognised the depth of her loss, the significance of her relationship with the deceased and the reasons behind her drug use. This previously established understanding and trust allowed her to maintain a connection that continued to be supportive despite her departure from the treatment facility, ongoing drug use and being in a difficult and vulnerable situation.

Elisabeth described a contrasting experience. She experienced that the professional who had supported both her and her intimate partner let her partner down by failing to provide the help he needed. After her partner died of an overdose, the professional's attempt to offer support was not welcomed. For example, the professional attended the funeral and sat beside her, which is a gesture Elisabeth found deeply unsettling. The professional's absence during her partner's life made it impossible for Elisabeth to welcome the bereavement support.

Erik relayed that negative experience with the support system dating back to his childhood led him to steadfastly reject outside assistance for his pain and loss. He was convinced that this refusal would remain unchanged: “My upbringing dictates that I refuse to let outsiders come and mess with my life again. The state has ruined my life enough. Stay the hell away, that's how I am, and that's how I will always be”.

Erik's view on representatives from the support system as outsiders, his belief that they will mess with his life and his statement that the state ruined his life clearly demonstrate a strong perception of a system of expert practices that is not compatible with or supportive of his conduct of everyday life.

The stories shared by our participants, encompassing both detrimental and beneficial interactions with the support system, illustrate how past experiences can be infectious or transferable; from one phase of life to another, from one support worker to another and across different organisational contexts. These statements confirm that professional help contexts do not operate in isolation but are interwoven with and influenced by other social contexts of expert practices. Thus, how well an initiative of support works seems to depend on the participants’ experiences from responses, events and activities in other help settings.

It is evident that establishing a reputation for trustworthy support is crucial, as it lays the foundation for whether or not helpers can assist bereaved individuals in coping with their bereavement after a drug-related death.

#### Offer supportive communication

Many participants articulated the need for their bereavement to be acknowledged and seen by others. This involved recognising the depth of their loss, understanding their bereavement and respecting their associated needs. Many participants recounted how their loss was overlooked or underestimated due to their relationship with the deceased not being acknowledged or their loss not being recognised as severe. Several expressed a desire to have someone to talk to about their loss. This is expressed in statements such as:

It might have been nice to have someone who would listen to you, ask if you were okay and whether there was anything you needed, things like that. And maybe to have been offered someone to talk to, a professional, if you needed it, but there was never any such offer. (Fia)

A person doesn't need massive help, but some periodic follow-up, like getting a phone call once in a while from some form of support system to check in on how things are going. (Frank)

Some expressed that they would have preferred a dialogue with someone knowledgeable about bereavement and well-versed in feelings and needs associated with loss. For others, it was crucial to talk to someone who understands the realities of drug use. Frida belonged to the latter group and reflects on this need:

Talking to others who don't relate to that life is difficult because they typically find it hard to understand. And when you want to talk about it, you need someone who gets what you’re saying. At least, that's how I am. If I were to talk about it at the time or during the grieving process, I would choose people who knew what it was about. It was too hard to encounter ignorance. Yes, maybe people don't know enough about [drug use], and that's a sign of health because it means they haven't had anything to do with it. And then maybe, it's probably also a part of this shame, right, that one has been in a drug environment, and it's not something to boast about. It's not an achievement in life, so to speak, to have been there. So, you choose to talk to the safe ones. (Frida)

Frida's statement highlights how a life of drug use is felt as unusual and shameful, and it is hard for others to comprehend and acknowledge it. Therefore, it is crucial to have support from someone who understands and recognises her conduct of everyday life when dealing with bereavement. The need to be understood in relation to their specific social life contexts can make it more difficult to seek help from services as the people providing these services are often perceived as not understanding or acknowledging their everyday life experiences.

Some participants pointed out that, in addition to seeking conversations about bereavement, it is nice to be able to talk about the deceased and the memories of life with them:

The most important thing for me has always been to stay positive. Reminiscing about the good, all the good stuff has really been the most important thing to me, not dwelling on all the negatives. Talk a lot about him, bring him into every context, keep him alive. (Fabian)

A few of the participants reflected upon that it was difficult for them to talk about the loss and that they might need gentle, well-meaning encouragement to talk about what is painful and hard to discuss. Dina reflects on this:

I’ve built up a defence. I don't like talking about someone's death. I don't like saying that he is dead. I’ve built a defence to protect myself, so when someone has tried, I just shut down. And I say, “not now, some other time, not now” – But then they stop trying, so it never leads to anything more. But that's not what I mean, really. They take me at my word, which doesn't always work well.

I often feel people shouldn't take my words quite literally when I say no, not now, next time, next time. But it doesn't help to insist: “Yes, now it matters”. But instead of: “Okay, yes, then we’ll do it next time”, you might try gently, maybe not go straight in, but continue talking gently and so later ask a little more that perhaps you don't dive straight into – what happened with that overdose, to be so direct, but you can gently ease into the subject. (Dina)

Dina's statement demonstrates the importance for helpers to tolerate rejection and continue to search for gentle and constructive approaches to engage in conversations about the loss. Her narrative suggests that talking about the death of her loved one is perceived as very different from what she is used to in her everyday life. Instead, she has, through her conduct of life, built up a defence where she shuts down. She expresses a desire to develop her skills in talking about the loss but points out that developing these skills takes time and patience. At the same time, she finds it difficult to be the agent of these changes herself; she needs helpers who address her troubles with a repeated, gentle approach to help her develop these new skills.

#### Foster tailored support

This theme concerns the significance of offering individually tailored support and engaging in dialogue with the bereaved persons about what constitutes good support and assistance for them.

The participants highlighted that people with heavy drug use represent a heterogeneous group, entrenched in different social practices, each with their unique experiences and needs concerning bereavement. An individual's needs and coping abilities may also change during their life course. Geir expressed it this way:

When people are actively using, they’re still in different places. Not everyone is equally out of it; they might have just relapsed, or they might have had their lives in order. They don't have to be equally damaged, and so you must talk to them and find out what they need to talk about. (Geir)

Several participants spoke of the difficulties they encountered due to others’, people in their network or professional helpers, non-acceptance of their drug use, people distancing themselves when drugs were used and being given an ultimatum to reach a state of remaining drug-free as a requirement for receiving help and support. An essential aspect of their desire for tailored support is the importance of not allowing drug use to obstruct the provision of individualised support. As exemplified by Gro, expressing a wish for helpers to communicate:

We won't stop you from using; it's there, and you can take it as soon as you like, but could you maybe just think about sitting and talking for a few minutes more with me and just try. I think that could go a long way. But often, as it has been with me too, you panic, I need drugs, and then you’re somewhat prepared that people will be like, “No, no drugs”, and then you want to get away even faster. So, I think, if someone had said to me like that: “Good heavens, you can use as much as you want”, quite early in the conversation, then I think already at that point I would have felt that I could relax a bit more because then I know that whatever happens here, the drugs are still there. (Gro)

Gro's desire for acceptance of her use in order to feel safe and not in a hurry during the help situation underscores the need for support that aligns with her conduct of everyday life. For her to be able to make use of the help offered, such as talking about the loss, it should not interfere with the strategies she typically uses in her daily life. Instead, she wants her drug use to be recognised as a significant aspect of her life, and she wants the loss to be addressed in ways that fit into her conduct of everyday life.

Another critical point raised by many participants is the necessity for enough time to process their loss and to approach and begin talking and coping with their loss gradually. They ask for support that is adaptable to the dynamic process, which may take time.

#### Promote support through companionship

The final theme underscores the importance of not being alone and of experiencing a sense of companionship and belonging. This fellowship does not need to focus on the loss or discuss what happened explicitly. Still, it enables the possibility of discussing the event if one wishes as well as simply sharing space and experiences together.

Low-threshold services, which allow individuals to come and go as they please, stay for as long as they need and choose to engage in conversations about death and bereavement if they wish, were particularly highlighted. In addition, some participants expressed appreciation of being part of a community that offers a break from their challenging feelings. For example, participants described positive experiences travelling together, finding solace in nature or doing activities together.

Companionship was also presented as beneficial for reducing the sense of being alone during a difficult time and for feeling acceptance, understanding and respect for the loss and the deceased without necessarily needing to talk about the loss and the bereavement. Hanne describes it like this:

I needed someone who was steady, rational and natural, maybe not having relational challenges themselves, but who was rational and generous. They were just there, and it was good. They didn't say much or do much, but I saw that they respected the deceased for who he was. (Hanne)

Companionship is also presented as vital for preventing drug use from becoming too intense and dangerous. Harald's statement illustrates: “For me, it was important to be with others because if I’d been alone, I would have been dead, stone dead. Yes, yes, then I would never have stopped using”.

The value of having others present and contributing to making drug use less dangerous highlights the importance of help that fits into their conduct of everyday lives. When heavy drug use is a central and valued activity in coping with bereavement, support that is adapted to this activity, such as someone helping to ensure safety, becomes essential assistance that fits into their everyday lives.

Summing up, although the findings point to ambivalence regarding the usefulness and the acceptance of support initiatives, the insights from the interview participants also point to some key dimensions that are important for strengthening responses to drug-related bereavement. In the following, we elaborate on this complex context for support provision, and we propose a three-tiered approach to improve bereavement support to individuals with dual experiences of heavy drug use and bereavement.

## Discussion

The participants’ insights regarding support needs emphasise life with drug use as a complex context for bereavement support provision. In the following, we discuss the findings through the lens of Dreier's sociality of human subjectivity (Dreier, 2011) and highlight some important aspects regarding the conduct of everyday life that influence support expectations and support received.

### Bereavement support and the conduct of everyday life

Due to the significant burdens that unnatural losses impose on the bereaved following unnatural deaths in general, it is strongly suggested that policymakers and clinicians working with people confronted with trauma and unnatural loss should be aware of bereavement needs (Djelantik et al., 2020). The results from this study showed that the participants generally had modest expectations of support. They were not sure whether the support would be helpful, or they would ask for modest forms of support, such as to be recognised as grievers or to receive a phone call now and then. Furthermore, the participants had limited concrete advice on how support should be arranged.

The ambivalence regarding the helpfulness and the content of support shown in this study can be a result of participants’ prior encounters with the professional helpers, as illustrated by Erik, who strongly stated that he was not available for help from professionals because of negative experiences in his childhood. This finding corroborates with findings from a study of families bereaved by substance-related death, where Valentine et al. (2016) found that poor experiences with professional helpers before death influenced the family's help-seeking behaviour after death. Following from the buffering theory of social support, a person's prior experiences of helpful support will influence the perceptions of support and inform the future expectations of support (Lakey & Orehek, 2011).

Negative experiences from prior help and support can also be understood considering the specific drug use-related social context of our participants. Dreier (2011) defines a social context as a particular material unit with its own regulations and expectations, providing positions and norms for activities and relationships within this context. Moving between different social contexts is part of everyday life, but for people who use illicit drugs, this can be particularly demanding as they have a lifestyle that lacks social recognition and are involved in activities that are illegitimate. As such, it can be more challenging for persons with heavy drug use to experience that other social contexts fit into their everyday life.

Research has shown that persons with heavy drug use experience both structural stigma and stigma from the professionals when they encounter the help system (Livingston et al., 2012; Van Boekel et al., 2013). Stigma typically involves labelling, such as being characterised as violent, manipulative or irresponsible, and the helpers report it to be emotionally challenging to work with this group and they can feel unsafe (Van Boekel et al., 2013). Hence, stigma relating to drug use can influence how people with heavy drug use approach support, their expectations of support and how they perceive themselves as legitimate support receivers. Furthermore, stigma can also influence the supporters’ behaviour. While stigma impacts support relationships, other aspects relating to the social context of drug use can also explain the complexity shown in the participant's reflections on what was helpful or unhelpful support. Dreier (2011) discusses how the effects of psychological interventions must be understood from a contextual perspective. An intervention developed within a specific therapeutic context, must be fitted to the context where the clients live their everyday life. Hence, expert practices must not overshadow or ignore the things that are important for the clients in their daily lives. In the present study, when Gro pointed to the importance of helpers recognising her need for drugs in order to accept help initiatives, tensions between the helping context and the client's context became evident. For a piece of advice or a help initiative to be perceived as helpful, it seems to have to fit into the clients’ conduct of everyday life.

Moving between different social contexts also implies adapting to and acquiring the different skills and positions inherent in the arrangements of the contexts. Some of the participants in this study described a lack of knowledge about the kind of services available, or they describe themselves in a position as unable to be helped. Having information about what services exist and the skills to approach them are perceived as preconditions for access to services in public bureaucratic systems (Schaffer & Wen-Hsien, 1975). Bureaucratic organisations are typically characterised by being rulebound and rigid, and access is given to those applicants who can demonstrate their eligibility for the services. Furthermore, the clients have to adapt to the rules for admission, the rules or standards for service delivery and the positions available for the clients within the system (Schaffer & Wen-hsien, 1975). For people who do drugs, these rules for distribution within the helping context can be difficult to adhere to and adjust to in their everyday practices.

One of the most common help interventions offered in bereavement support is to talk about the loss and the feelings related to the loss with a therapist or, eventually, with a group of peers (Cartwright, 2024; Holte Kofod, 2017; Madsen, 2014). Support from a psychiatrist is also the type of support most frequently mentioned when persons bereaved after a drug-related death report the type of support they have missed most (Kalsås et al., 2023), also signalling the dominating perceptions of bereavement support in the societal discourse. When the participants in this study were unsure as to whether they would have accepted support initiatives or not, this can indicate that the dominating practices of addressing grief needs do not fit into their conduct of everyday lives. Addressing grief through therapeutic talk can contradict the conduct of everyday life wherein talking about feelings is not common (see also Selseng et al., 2023) and, if it requires abstaining from drug use, interferes with strategies that are perceived as important and meaningful. The analysis shows that some expressed a desire to develop skills to talk about their grief but asked for an understanding that this is a different activity that takes time to develop competence in.

The results of the present study also highlight positive experiences with the support system. In particular, contact with low-threshold services and residential treatment facilities were described as an important source of support. The social contexts of these services are probably more flexible and less bureaucratic, thus providing both the helpers and the clients with positions that can be more dynamic, and less standardised, increasing the possibilities for help initiatives to be accepted and adapted by the clients into their everyday life context. For example, this is shown by Dina who continued the contact with some of the staff members after she had left the treatment facility. Dina described how this contact became important for her bereavement process and particularly pointed to how she felt understood and recognised by the staff members. This highlights the importance of helpers’ knowledge and affiliations with the social context where the clients live their lives. Probably, helpers working within the drug use services will have more insights into their clients’ everyday life practices, and the norms and expectations within the social context of drug use. This contextual knowledge can increase the possibilities for their interventions to fit and for them to be accepted by the clients.

However, research has pointed to a lack of knowledge on bereavement among workers in drug use services (Reime et al., 2024a; Valentine et al., 2018). A relevant question is how well these helpers are equipped to address bereavement issues among their clients and recognise and provide help to the clients not only as individuals with heavy drug use, but also as grievers (Selseng et al., 2023). Moreover, based on the assumption that people are always situated in various social contexts, it is also relevant to question how helpers within the bereavement field who are situated in other contexts with other norms and expectations can become relevant and accessible for clients within the social context of drug use.

#### A three-tiered approach to improve bereavement support

With the aim of stimulating and contributing to discussions on how both drug use services and grief services can offer help that fits into the conduct of everyday life of clients who engage in heavy drug use, we present examples of bereavement support using a three-tiered approach (Killikelly et al., 2021). Because each bereaved individual will have unique needs, it is essential to provide the bereaved persons with the right help at the right time (Killikelly et al., 2021). Most bereaved persons will adjust to life after the loss of a significant person, either alone or with support from their closest network (general support). However, some persons will need additional support (selective support) and a few will need specialist treatment (indicated support) (Killikelly et al., 2021). For bereaved persons with a low risk for bereavement complications, general support (such as information, peer support, self-help and support from professionals who do not specialise in bereavement) is often sufficient. For persons with more risk of developing complicated grief reactions (e.g., those who have experienced a sudden and unnatural death), selective support, such as psycho-social follow-up, can be appropriate. For bereaved persons with symptoms of complicated grief, more specialised services will be needed that are provided by trained professionals (Killikelly et al., 2021).

Insights from the participants in this study primarily highlight the importance of support initiatives anchored on the lower levels of the three-tiered approach, and they call for support initiatives that consider the particular social context where the person using drugs is situated, and that can be useable within this context. First of all, it stands out as essential to be met as bereaved individuals and not merely individuals with heavy drug use. Here, the different social contexts that are a part of the bereaved persons’ everyday life can play a powerful role. To be seen and recognised as bereaved individuals is also dependent upon the helper's confidence in addressing these issues when they encounter clients with dual experiences of heavy drug use and bereavement. Research has pointed to a general lack among people working in the low-threshold services of knowledge on bereavement (Reime et al., 2024a; Valentine et al., 2018). Simultaneously, helpers working with psycho-social follow-up describe a lack of competence in meeting bereaved persons who use drugs (Løseth et al., 2023b). A lack of knowledge on the context of where people live their everyday lives makes the helpers unconfident and can impede help provision or lead to help that is of no use (Dreier, 2011). For example, helpers in low-threshold services may be reluctant to address clients’ bereavement experiences because they are afraid that this can lead to a relapse (Reime et al., 2024a). When helpers avoid addressing bereavement among clients, this can be (mis)understood as a lack of recognition. Hence, to increase low threshold services, general competence in bereavement can be important both to facilitate recognition and for increasing the quality of support provided at the lower levels. Lack of knowledge on bereavement care has been described as a general deficit among service providers working with other groups of bereaved persons as well, such as among those practitioners working in palliative care (Fan & Lin, 2022). Hence, insights from this study support other studies, addressing the need for an increased focus on provider education and training (Fan & Lin, 2022; Løseth et al., 2023a; Totland et al., 2024).

This study has shown that bereaved persons with a dual experience of heavy drug use over time, to a small degree, have been offered help despite being at risk for developing complicated grief reactions (Parisi et al., 2019; Wojtkowiak et al., 2019). It is therefore vital to consider how to develop selective interventions that are adjusted to this group of bereaved persons’ needs for support and their everyday conduct of life. The participants do not expect (or desire) services that are comprehensive; rather, they aim for services that are flexible and available. The desires expressed appear to be similar to those for substance use services in general. There is a need for flexibility, cohesive services and to be seen and treated as individuals (for an example, see Hansen et al., 2020). One example of such interventions can be psycho-social services that are given in the form of outreach and that provide flexible follow-ups over time, which are also recommended in the Norwegian guidelines on social support in case of sudden and unnatural deaths (Norwegian Directorate of Health, 2016).

Persons with heavy drug use typically have experienced many losses and the deaths of close ones (Kenny et al., 2022). However, some periods or life situations can be more likely to trigger feelings related to bereavement than others. To be in a treatment situation can be one example of a situation that can strengthen or reinforce the bereavement experiences. Such periods of abstinence can trigger emotions that have earlier been suppressed by drugs (Selseng et al., 2023). It is therefore important that helpers working in drug use services acknowledge the clients’ bereavement experiences and have sufficient knowledge to help the clients process their losses and cope with the related feelings. This includes knowledge of how to reach bereaved individuals who use drugs, what bereavement support can be helpful, and the personal competence to show respect and consideration for the bereaved individuals (Furr & Hunsucker, 2022; Totland et al., 2024).

The high risk for bereavement complications that are found among bereaved persons with heavy drug use over time (Parisi et al., 2019; Wojtkowiak et al., 2019) also calls for attention to the third level of the tiered approach to bereavement support. It is crucial that helpers who relate to the bereaved persons at the other levels, through low threshold services or by providing psycho-social support, can recognise signals of complicated grief and can help provide access to adequate support.

The present study concerned the experiences of 13 individuals with dual experiences of loss and drug use. Due to the relatively small number of participants, the diversity in the participants’ current drug status (e.g., in treatment, actively using or in recovery process) and the anchoring in the Norwegian welfare state, some caution must be taken regarding the transferability of the study's results. However, the study provides unique insights from the perspectives of individuals with lived experience in a scarcely researched area that can inform both practice and further research. Considering the critical nature of a bereavement situation managed through heavy drug use and considering the potential for how the experience of loss can facilitate change in one's life (Selseng et al., 2023), this is a life situation that necessitates further knowledge. There is a need for more research on how to enhance contextualised support for bereaved individuals with heavy drug use, during both the acute period following a loss and later on in the bereavement process, which acknowledges and fits into their conduct of everyday life.
